# 2239. Antibiotic Sensitivity Spectrum Of Acute Bacterial Cholangitis: Trends From A Tertiary Referral Center In Pakistan

**DOI:** 10.1093/ofid/ofad500.1861

**Published:** 2023-11-27

**Authors:** Muhammad Abdurrahman Butt, Maahin Manzoor Khan, Myyra Omar, Mehwish Rafique, Maaz Badshah, Mohammad Salih, Muslim Atiq

**Affiliations:** Shifa College of Medicine, Lahore, Punjab, Pakistan; Shifa College of Medicine, Lahore, Punjab, Pakistan; Shifa College of Medicine, Lahore, Punjab, Pakistan; Shifa International Hospital, Islamabad, Islamabad, Pakistan; Shifa international hospital, Islamabad, Islamabad, Pakistan; Shifa International Hospitals Ltd, Islamabad, Islamabad, Islamabad, Pakistan; Shifa International Hospital, Islamabad, Islamabad, Pakistan

## Abstract

**Background:**

Acute bacterial cholangitis can result in significant morbidity. Aggressive antibiotic therapy and prompt endoscopic or percutaneous biliary decompression are key to improving outcomes. However, evolving antimicrobial resistance patterns pose a significant challenge to treating these infections especially when deciding about empiric therapy.

**Methods:**

The study was conducted at Shifa International Hospital in Islamabad. All the patients with the diagnosis of Acute bacterial cholangitis between July 2016 and June 2022 were included. The study was approved by institutional IRB. Data was analyzed using the Pearson Chi-Squared test.

**Results:**

In this study, a total of 144 consecutive patients were included, out of which 51 (35.4%) had a growth on blood cultures. The most commonly identified organism was E. Coli found in 30 (58.8%) followed by Pseudomonas in 7 (4.9%), Klebsiella pneumonia in 6 (11.8%), Proteus in 3 (5.9%), and Enterococcus in 1 (2.0%) patients. Other organisms were found in 15 (29.4%) patients. Polymicrobial infection was found in 12 (23.5%) patients. Out of the blood cultures with growth, 10 (19.607%) were identified as multi-drug resistant (MDR) species, while 19 (37.25%) were identified as extended-spectrum beta-lactamase (ESBL) species. Patients who have undergone prior biliary intervention (ERCP, PTBD or both) were more likely to be infected with E.Coli (MDR)(P=0.026), Proteus(P=0.033), or polymicrobial strains (P=0.040). Antibiotic sensitivity was 10.9% for Ceftazidime, 29.5% for Piperacillin/Tazobactam, 31.3% for Ertapenem, 59.4% for Colistin, 71.7% for Meropenem and 72.9% for Imipenem.

Organisms Identified on Culture. (Table 1)

**Figure:**
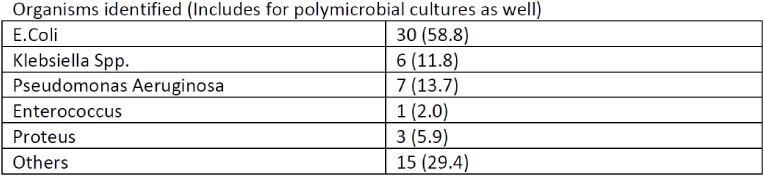

Organisms Identified on Culture. (Table 1)

Antibiotic Sensitivity Spectrum. (Graph 1)

**Figure:**
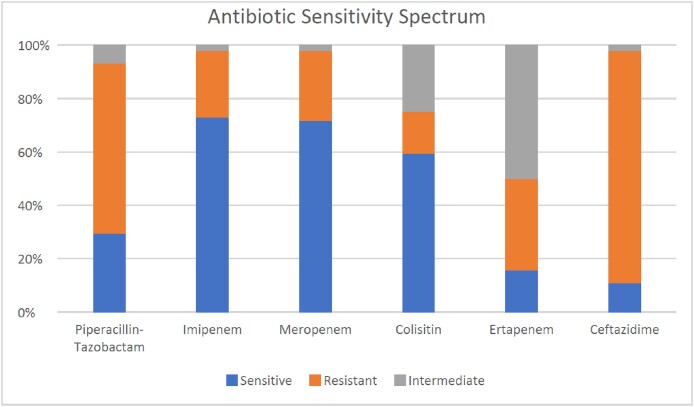

Antibiotic Sensitivity Spectrum. (Graph 1)

**Conclusion:**

Antibiotic resistance poses a major challenge in treating Acute Bacterial Cholangitis. The most effective antibiotics found for treating this condition in our population were Meropenem, Colistin, and Imipenem, while high resistance patterns were observed for Ertapenem, Ceftazidime, and Piperacillin/Tazobactam. A balance in prescribing broad-spectrum antibiotics and avoiding their use without definitive evidence of infection is crucial in mitigating the growing threat of antimicrobial resistance in cholangitis.

**Disclosures:**

**All Authors**: No reported disclosures

